# Regional differences in physicians’ behavior and factors influencing the intensity of PCSK9 inhibitor therapy with alirocumab: a subanalysis of the ODYSSEY APPRISE study

**DOI:** 10.3389/fcvm.2023.1206551

**Published:** 2023-06-19

**Authors:** Maciej Banach, Joanna Lewek, Kaja Pol, Daniel Rabczenko, Serban M. Balanescu, Vladimir Blaha, Richard Ceska, Piotr Jankowski, Stanisław Surma, Genovefa Kolovou, Evangelos Liberopoulos, Florin Mitu, Magda Mitu, Franjo Husam Naji, Gyorgy Paragh, Magdalena Popławska, Michal Vrablik, Daniel Pella

**Affiliations:** ^1^ Department of Preventive Cardiology and Lipidology, Medical University of Lodz (MUL), Lodz, Poland; ^2^ Department of Cardiology and Congenital Diseases of Adults, Polish Mother's Memorial Hospital Research Institute (PMMHRI), Lodz, Poland; ^3^ Cardiovascular Research Centre, University of Zielona Gora, Zielona Gora, Poland; ^4^ Ciccarone Center for the Prevention of Cardiovascular Disease, Johns Hopkins University School of Medicine, Baltimore, MD, United States; ^5^ Sanofi, Bridgewater, NJ, United States; ^6^ Department of Population Health Monitoring and Analysis, National Institute of Public Health NIH-National Research Institute, Warsaw, Poland; ^7^ Department of Cardiology, Elias Emergency University Hospital, Carol Davila University of Medicine and Pharmacy, Bucharest, Romania; ^8^ The 3rd Department of Internal Medicine—Metabolic Care and Gerontology, Charles University and University Hospital in Hradec Králové, Hradec Králové, Czechia; ^9^ 3rd Department of Medicine—Department of Endocrinology and Metabolism of the First Faculty of Medicine, Charles University and General University Hospital, Prague, Czechia; ^10^ Department of Internal Medicine and Geriatric Cardiology, Center of Postgraduate Medical Education, Warsaw, Poland; ^11^ Department of Epidemiology and Health Promotion, School of Public Health, Center of Postgraduate Medical Education, Warsaw, Poland; ^12^ Faculty of Medical Sciences in Katowice, Medical University of Silesia, Katowice, Poland; ^13^ Cardiometabolic Center, Metropolitan Hospital, Piraeus, Greece; ^14^ First Department of Propaedeutic Internal Medicine, Medical School, National and Kapodistrian University of Athens, Laiko General Hospital, Athens, Greece; ^15^ Department of Medical Specialties I, “Grigore T. Popa”, University of Medicine and Pharmacy, Iasi, Romania; ^16^ Cardiovascular Rehabilitation Clinic, Clinical Rehabilitation Hospital, Iasi, Romania; ^17^ University Clinical Center Maribor, Maribor, Slovenia; ^18^ Division of Metabolism, Department of Internal Medicine, Faculty of Medicine, University of Debrecen, Debrecen, Hungary; ^19^ Sanofi, Warsaw, Poland; ^20^ 2nd Department of Cardiology of the East Slovak Institute of Cardiovascular Disease and Faculty of Medicine, PJ Safarik University, Kosice, Slovakia

**Keywords:** alirocumab, LDL-C, PCKS9 inhibition, therapy goals, therapy intensity

## Abstract

**Background:**

Despite better accessibility of the effective lipid-lowering therapies, only about 20% of patients at very high cardiovascular risk achieve the low-density lipoprotein cholesterol (LDL-C) goals. There is a large disparity between European countries with worse results observed for the Central and Eastern Europe (CEE) patients. One of the main reasons for this ineffectiveness is therapeutic inertia related to the limited access to appropriate therapy and suitable dosage intensity. Thus, we aimed to compare the differences in physicians’ therapeutic decisions on alirocumab dose selection, and factors affecting these in CEE countries vs. other countries included in the ODYSSEY APPRISE study.

**Methods:**

ODYSSEY APPRISE was a prospective, single-arm, phase 3b open-label (≥12 weeks to ≤30 months) study with alirocumab. Patients received 75 or 150 mg of alirocumab every 2 weeks, with dose adjustment during the study based on physician's judgment. The CEE group in the study included Czechia, Greece, Hungary, Poland, Romania, Slovakia, and Slovenia, which we compared with the other nine European countries (Austria, Belgium, Denmark, Finland, France, Germany, Italy, Spain, and Switzerland) plus Canada.

**Results:**

A total of 921 patients on alirocumab were involved [modified intention-to-treat (mITT) analysis], including 114 (12.4%) subjects from CEE countries. Therapy in CEE vs. other countries was numerically more frequently started with lower alirocumab dose (75 mg) at the first visit (74.6 vs. 68%, *p* = 0.16). Since week 36, the higher dose was predominantly used in CEE patients (150 mg dose in 51.6% patients), which was maintained by the end of the study. Altogether, alirocumab dose was significantly more often increased by CEE physicians (54.1 vs. 39.9%, *p* = 0.013). Therefore, more patients achieved LDL-C goal at the end of the study (<55 mg/dl/1.4 mmol/L and 50% reduction of LDL-C: 32.5% vs. 28.8%). The only factor significantly influencing the decision on dose of alirocumab was LDL-C level for both countries’ groups (CEE: 199.2 vs. 175.3 mg/dl; *p* = 0.019; other: 205.9 vs. 171.6 mg/dl; *p* < 0.001, for 150 and 75 mg of alirocumab, respectively) which was also confirmed in multivariable analysis (OR = 1.10; 95% CI: 1.07–1.13).

**Conclusions:**

Despite larger unmet needs and regional disparities in LDL-C targets achievement in CEE countries, more physicians in this region tend to use the higher dose of alirocumab, they are more prone to increase the dose, which is associated with a higher proportion of patients reaching LDL-C goals. The only factor that significantly influences decision whether to increase or decrease the dose of alirocumab is LDL-C level.

## Introduction

Atherosclerotic cardiovascular disease (ASCVD) is the leading cause of morbidity and mortality worldwide (1, 2). In 2019, the number of patients with cardiovascular diseases worldwide was 523 million, while the number of deaths due to these diseases reached 18.6 million (2, 3). In 2017, the number of patients with coronary artery disease (CAD) worldwide reached 126 million (1.72% of the world population), and it is estimated to increase every year. Worldwide, CAD caused nine million deaths in 2017, making the disease the leading cause of death (4). The incidence of stroke is also a significant problem. In 2019, the number of patients with stroke worldwide was 101 million, while the number of deaths due to stroke was 6.55 million (5). Peripheral arterial disease (PAD) is also a widespread disease. In 2019, the number of patients with PAD worldwide was 113 million, and the disease caused 74.1 thousand deaths (3).

The most important risk factor for ASCVD is hypercholesterolemia (2). Prevention and effective lipid-lowering treatment is the most effective therapy to prevent ASCVD (6). Every 1% reduction in low-density lipoprotein cholesterol (LDL-C) is associated with a reduction in cardiovascular risk of approximately 1% (7). After 5 years, the risk is reduced by about 20%–25%, and after 40 years even by 50%–55% (risk reduction in every second patient) for each mmol/L of lowered LDL-C (8). Despite the proven effectiveness of lipid-lowering treatment in the primary and secondary prevention of ASCVD, only every third patient in Europe achieves the therapeutic goal (irrespectively on the risk), i.e., only 18% in Europe reach the goal for very high CVD risk patients (<55 mg/ dl/ < 1.4 mmol/l) in comparison to only 13% in the countries of Central and Eastern Europe (CEE) (9, 10). The picture is even more challengeable when we add that less than 10% of patients at extremely high cardiovascular risk are within the therapeutic target (<40 mg/dl/ < 1 mmol/L) (9, 10). Patients’ nonadherence as well as therapeutic inertia associated with lack of suitable therapy and dose intensity are among the main causes of this ineffectiveness (6).

Thus, we decided to compare the efficacy differences in LDL-C target achievement and the differences in physicians’ therapeutic behaviors on dose selection of the proprotein convertase subtilisin/kexin 9 (PCSK9) inhibitor—alirocumab and factors influencing these in CEE countries vs. other countries that participated in the ODYSSEY APPRISE study.

## Methods

ODYSSEY APPRISE (NCT02476006) was a prospective, single-arm, phase 3b open-label (≥12 weeks to ≤30 months) study with alirocumab in a real-life setting (11). It was designed to obtain data regarding safety and efficacy among high cardiovascular risk patients who were not adequately controlled by lipid-lowering therapy due to severe hypercholesterolemia (11, 12). Patients were enrolled between 23 June 2015 and 12 April 2019. A complete list of investigators as well as study sites is described in detail elsewhere (11). In each country, when alirocumab became commercially available (i.e., accessible to the patient in accordance with each nation's regulations) and reimbursed, patient recruitment was ended. Once the patient finished the required minimum of 12 weeks of study medication, study treatment was been shifted to the commercial product.

The study was conducted in accordance with the principles of the Declaration of Helsinki and the International Conference on Harmonization Guidelines for Good Clinical Practice. The trial protocol was approved by local authorities, appropriate independent ethics committee, or institutional review board at each participating study center. Written informed consent was obtained from all participants before study entry (11).

### Study design

After a screening period of up to 3 weeks, patients received 75 or 150 mg subcutaneous alirocumab every 2 weeks. The starting dose was based on basic patient characteristics and treatment LDL-C goals. The dose was adjusted from 75 to 150 mg twice a week based on the physician's judgment. All the patients were on stable treatment with maximally tolerated statin and other lipid-lowering drugs. Maximally tolerated statin therapy was defined as 20 or 40 mg/day rosuvastatin, 40 or 80 mg/day atorvastatin, or 80 mg/day simvastatin therapy for more than 1 year. In case of intolerance of such a dose, patients were permitted to be treated with a lower dosage based on the physician's judgment. The other statin regimen was also allowed in the documented exceptional cases. However, the dose and regimen of lipid-lowering therapy was meant to be stable throughout the whole study duration. The modification of therapy was allowed only in exceptional cases after careful clinical judgment. The duration of open-label treatment with alirocumab was a minimum of 12 weeks and a maximum of 30 months.

### Study population

Patients included in the study were aged >18 years and were not adequately controlled for their heterozygous familial hypercholesterolemia (HeFH) or coronary heart disease or its equivalent. The detailed characteristics of studied population were previously described in the study protocol (11). The study was conducted in Austria, Belgium, Canada, Czechia, Denmark, Finland, France, Germany, Greece, Hungary, Italy, Poland, Romania, Slovakia, Slovenia, Spain, and Switzerland. The subanalysis of the ODYSSEY APPRISE assessing the regional differences in physicians’ behavior and factors influencing the intensity of PCSK9 inhibitor therapy with alirocumab was not prespecified in the study protocol. For the purposes of the analysis, the CEE countries group included Czechia, Greece, Hungary, Poland, Romania, Slovakia, and Slovenia, which were compared with the other nine European countries (Austria, Belgium, Denmark, Finland, France, Germany, Italy, Spain, and Switzerland) plus Canada.

### Study endpoints

The targeted LDL-C was LDL-C < 55 mg/dl (1.4 mmol/L) and/or a 50% LDL-C reduction for all patients. There was also the combined goal of LDL-C < 55 mg/dl and 50% LDL-C reduction. Those LDL-C goals, based on European 2019 guidelines (13) were not prespecified in the study protocol. The Friedewald formula was used to calculate the level of LDL-C at any analysis time (14). However, in case of triglycerides higher than 4.5 mmol/L (400 mg/dl), the LDL-C value was not calculated and, therefore, was not included in the final analysis.

### Statistical analysis

Descriptive statistics for continuous variables are presented as mean and as frequency and percentages for categorical variables. Comparison between groups of patients from CEE and other countries were done using Wilcoxon rank sum test (continuous variables) and chi-square test (categorical variables). Computations were performed using R.4.0.5 statistical software. Statistical significance was defined as two-sided *p* < 0.05.

## Results

### Baseline characteristics

A total of 921 patients on alirocumab were included (mITT analysis). Among them, 114 subjects (12.4%) were from the CEE countries. Baseline characteristics of the population studied are shown in Table 1. In the CEE group, there were more females than in the other investigated countries (46.5% vs. 35.9%, *p* = 0.029), as well as higher prevalence of never-smoking patients (55.3% vs. 38.4%, *p* = 0.001). All patients from CEE group were of White/Caucasian race. No differences in the CVD risk of the investigated patients were observed (Table 1). The baseline level of LDL-C did not significantly differ between CEE group and other countries (mean: 181.4 vs. 182.6 mg/dl).

**Table 1:** Clinical characteristics and smoking status.

Characteristic	CEE, *N* = 114^a^	Other, *N* = 807^a^	*p*-value^b^
Sex			0.029
Female	53 (46.5%)	290 (35.9%)	
Male	61 (53.5%)	517 (64.1%)	
Race			0.062
Other	0 (0.0%)	25 (3.1%)	
White/Caucasian	114 (100.0%)	782 (96.9%)	
Age group (years)			0.60
<50	36 (31.6%)	213 (26.4%)	
50–58	25 (21.9%)	195 (24.2%)	
58–65	27 (23.7%)	182 (22.6%)	
≥65	26 (22.8%)	217 (26.9%)	
Smoking status			0.001
Current	18 (15.8%)	131 (16.2%)	
Former	33 (28.9%)	366 (45.4%)	
Never	63 (55.3%)	310 (38.4%)	
Groups of CVD risks			
Risk A^c^			0.24
	67 (58.8%)	520 (64.4%)	
Yes	47 (41.2%)	287 (35.6%)	
Risk B			0.88
	81 (71.1%)	579 (71.7%)	
Yes	33 (28.9%)	228 (28.3%)	
Risk C			0.34
	84 (73.7%)	559 (69.3%)	
Yes	30 (26.3%)	248 (30.7%)	
Risk D			0.67
	91 (79.8%)	630 (78.1%)	
Yes	23 (20.2%)	177 (21.9%)	
Risk E			0.12
	95 (83.3%)	620 (76.8%)	
Yes	19 (16.7%)	187 (23.2%)	

CEE, Central and Eastern Europe; CHD, coronary heart disease; CVD, cardiovascular disease; HeFH, heterozygous familial hypercholesterolemia; LDL-C, low-density lipoprotein cholesterol.

^a^ *n* (%).

^b^ Pearson's chi-squared test or Fisher's exact test.

^c^ Risk A: patients with HeFH with LDL-C ≥ 4.1 mmol/L (160 mg/dl) despite treatment; Risk B: patients with HeFH with LDL-C ≥ 3.4 mmol/L (130 mg/dl) despite treatment, and ≥2 CV risk factors; Risk C: patients with HeFH with LDL-C ≥ 3.4 mmol/L (130 mg/dl) despite treatment, and established CHD or other CVD, diabetes, or a family history of CHD; Risk D: non-FH patients with established CHD or other CVD, and with LDL-C ≥ 3.4 mmol/L (130 mg/dl); Risk E: patients with progressive CVD (coronary artery disease or peripheral arterial occlusive disease or cerebrovascular disease as documented clinically or by imaging techniques, with a subsequent CV event despite treatment) and LDL-C ≥ 2.6 mmol/L (100 mg/dl).

### Concomitant treatment

Statins were administered, respectively, in 99.1% and 97.9% of patients in CEE and other countries groups. The frequency of fibrates and bile acid sequestrant therapy was also similar between groups (19.3% vs. 19.2% and 10.5% vs. 17.2%). Patients from CEE group were significantly less frequently treated with ezetimibe (47.4% vs. 60.2%; *p* = 0.009), niacin (0.0% vs. 7.25; *p* = 0.003), and omega−3 fatty acids (3.5% vs. 8.9%; *p* = 0.049) (Table 2).

**Table 2:** Concomitant therapies applied in the study.

Characteristics	CEE, *N* = 114^a^	Other, *N* = 807^a^	*p*-value^b^
HMG CoA reductases inhibitors (Statin)			0.71
	1 (0.9%)	17 (2.1%)	
Yes	113 (99.1%)	790 (97.9%)	
Fibrates			0.98
	92 (80.7%)	652 (80.8%)	
Yes	22 (19.3%)	155 (19.2%)	
Bile acid sequestrant			0.071
	102 (89.5%)	668 (82.8%)	
Yes	12 (10.5%)	139 (17.2%)	
Cholesterol absorption inhibitor			0.009
	60 (52.6%)	321 (39.8%)	
Yes	54 (47.4%)	486 (60.2%)	
Nicotine acid and derivatives (Niacin)			0.003
	114 (100.0%)	749 (92.8%)	
Yes	0 (0.0%)	58 (7.2%)	
Omega 3 fatty acid (>= 1,000 mg/day)			0.049
	110 (96.5%)	735 (91.1%)	
Yes	4 (3.5%)	72 (8.9%)	
Other			0.001
	114 (100.0%)	740 (91.7%)	
Yes	0 (0.0%)	67 (8.3%)	

^a^ *n* (%).

^b^ Fisher's exact test; Pearson's Chi-squared test.

### Treatment goal

**Figure 1:**
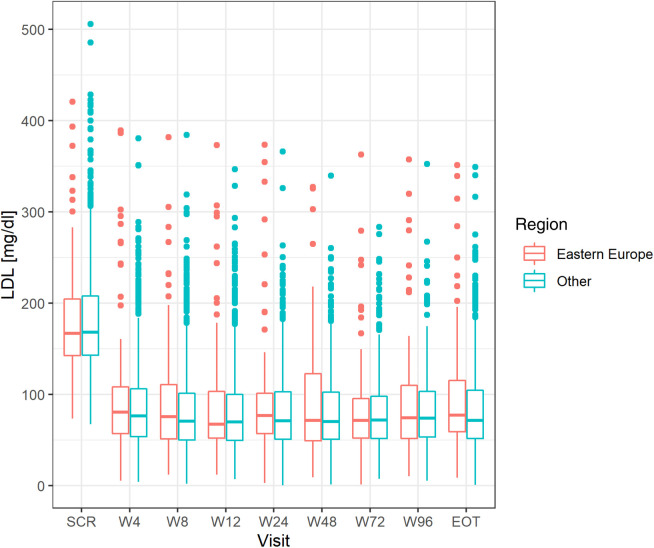
Differences in LDL level at all study points between the CEE group and other countries. LDL, low-density lipoprotein; CEE, Central and Eastern Europe.

**Figure 2:**
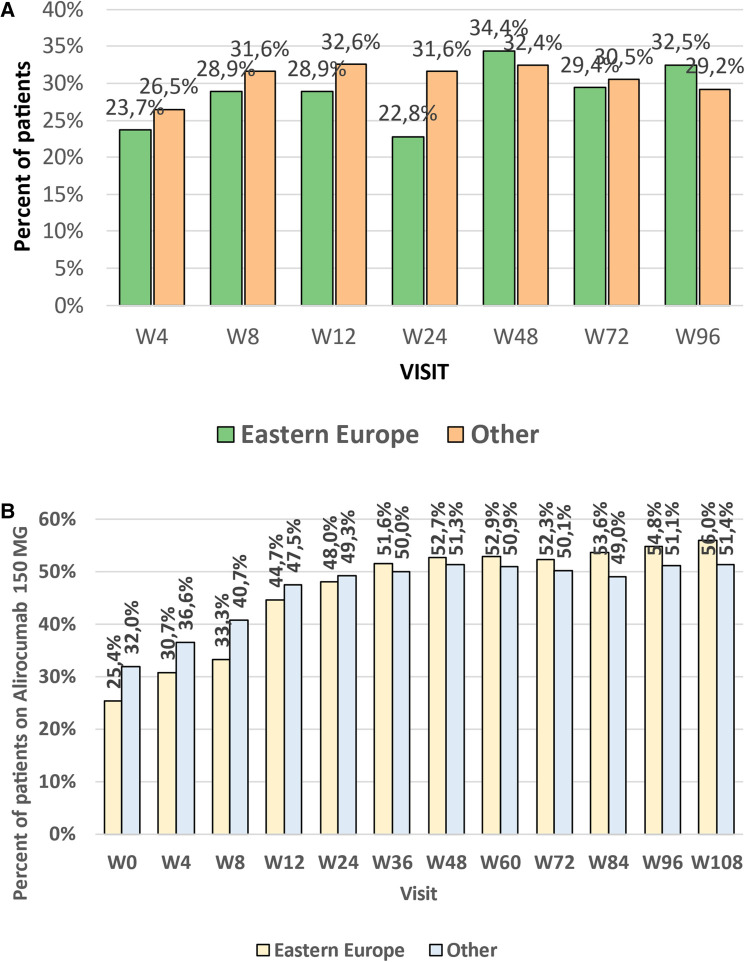
(**A**) Percent of patients being on combined LDL-C target (<55 mg/dl and 50% reduction) in weeks 4–96 and trends line for the efficacy changes in time. (**B**) Percent of patients treated with high dose of alirocumab (150 mg every 2 weeks) at the subsequent study points (week 4 to week 108) in the CEE and other countries groups, and trends line for the percentage changes over time. LDL-C, low-density lipoprotein cholesterol; CEE, Central and Eastern Europe.

There were no differences in the mean achieved level of LDL-C between the groups (Figure 1) in all investigated study points (week 4–96). Numerically less patients in CEE vs. other countries achieved LDL-C levels <55 mg/dl (1.4 mmol/L) at week 4–24 (from 22.8% to 28.9% vs. 26.5% to 32.6%), and numerically more at week 48 (34.4% vs. 32.4%) and 96 (32.5% vs. 29.2%). The number of patients that met both LDL-C level <55 mg/dl (1.4 mmol/L) and 50% LDL-C reduction showed similar trend, and at the end of the study 32.5 and 28.8% (*p* = 0.53) met these criteria in CEE vs. other countries group, respectively (Figure 2A).

### Dose adjustment

The initial dose of alirocumab during the first visit proposed by physicians in CEE countries, in comparison to that in other investigated countries, was statistically significantly lower. The physicians in the CEE group started therapy numerically more often with an alirocumab dose of 75 mg every 2 weeks [week (W) 0: 74.6 vs. 68%, *p* = 0.16]. Such a dose was maintained till week 24 (52.0 vs. 50.7%, *p* = 0.81). However, that trend was changed since week 36 when the higher alirocumab dose (150 mg Q2W) was more frequently applied in CEE vs. other countries’ patients (W36: 150 mg Q2W dose in 51.6% patients; *p* = 0.78), which was maintained by the end of the study and was numerically higher than in the group of other countries (W108: 56.0% vs. 51.4%, *p* = 0.46) (Figure 2B).

Altogether, physicians from CEE group significantly more often decided to increase alirocumab dose (54.1% vs. 39.9%, *p* = 0.013), which at the end of the study resulted with the higher ratio of patients who achieved recommended level of LDL-C.

The only factor that significantly influenced the decision on the starting dose of alirocumab was LDL-C level, which was significantly higher in patients with the starting dose of 150 mg Q2W vs. 75 mg Q2W in both country groups (CEE: 199.2 vs. 175.3 mg/dl; *p* = 0.019, other: 205.9 vs. 171.6 mg/dl; *p* < 0.001). This was also confirmed in the multivariable analysis, each LDL-C increase by 10 mg/dl was associated with 10% increase of the chance of administration of alirocumab at the dose of 150 mg/dl Q2W for the whole cohort (OR = 1.10; 95% CI: 1.07–0.1.13), with significant results for the other countries group, and with only a trend toward significance in the CEE group (OR = 1.06; 95%CI: 0.99–1.14; *p* = 0.093) (Table 3).

**Table 3:** Factors influencing decision on alirocumab dose administration 150 mg based on the multivariate analysis. Analyses for whole dataset and by regions.**.**

	Both regions			CEE countries			Other countries		
Characteristic	OR	95% CI	*p*-value	OR	95% CI	*p*-value	OR	95% CI	*p*-value
Region									
Eastern Europe	1.00	—							
Other	1.40	0.89–2.26	0.160						
LDL/10units increase	1.10	1.07–1.13	<0.001	1.06	0.99–1.14	0.093	1.10	1.07–1.14	<0.001
Age (years)									
<50	1.00	—		1.00	—		1.00	—	
50–58	1.02	0.68–1.53	0.905	0.59	0.16–1.95	0.398	1.10	0.72–1.69	0.662
58–65	1.34	0.89–2.02	0.159	1.21	0.40–3.66	0.734	1.37	0.88–2.13	0.159
≥65	0.82	0.54–1.24	0.344	0.45	0.11–1.63	0.243	0.88	0.57–1.37	0.578
Sex									
Female	1.00	—		1.00	—		1.00	—	
Male	1.19	0.87–1.64	0.270	1.08	0.44–2.72	0.863	1.21	0.87–1.69	0.269

OR, odds ratio; CI, confidence interval; LDL, low-density lipoprotein.

## Discussion

This *post-hoc* subanalysis of the ODYSSEY APPRAISE study in patients at high or very high risk of future cardiovascular events or with severe hypercholesterolemia showed that the ratio of patients being on the therapeutic goal of LDL is similar between CCE and other countries. This was achieved thanks to the drugs dose adjustment by healthcare professionals. The results of the study also clearly showed that the intensity of the lipid-lowering therapy is a critical factor to have the patients on LDL-C goals; therefore, one should start early to meet the therapeutic algorithm “the earlier the better” and “the lower the better” (15, 16). To the best of our knowledge, it is the first study carefully evaluating the important aspect of physicians’ behavior that may help reduce the risk of the physician inertia.

Even though most of the countries included in the CEE group are high-risk countries and most of the other group countries are moderate- or low-risk countries (17), in our cohort, there were no differences in the cardiovascular risk between the CEE group and other countries. The lack of differences between groups facilitates comparisons. It may have been related with the fact that participants of clinical studies, especially phase 3b that reflects real-life settings (11), are those willing to be cured; therefore, their cardiovascular risk may be lower. The usage of lipid-lowering therapies was also higher in our study than in other populations. In the Da Vinci study, a cross-sectional observational study of primary and secondary prevention patients from CEE countries, 92% of patients received statins (9, 10). Similarly, in other published data from CCE group, the prevalence of statin use was 92% in the TERCET Registry with 38% intensive statin therapy (18). In the longitudinal study from Czechia, statins were used in 79% patients (19). The same pattern was observed for ezetimibe, which in RWE is used in no more than 15%–20% (20), whereas in the ODYSSEY APPRISE, its use was very high, even >60% in the other countries, the level we indeed should aim for in our clinical practice.

The possibility to use PCSK9 inhibitor allowed us to achieve LDL-C goal in a satisfactory percentage of the population. Otherwise, available data suggest that LDL-C goal attainment in CEE countries is low and divergent for different countries ranging from 11% in Ukraine to 32% in Poland (10). These variations may be provoked by differences in lifestyle, various healthcare systems, socioeconomic factors, differing statin availability at all doses, unique prescription requirements (for example, in some countries, only specialists can prescribe ezetimibe), and constrained reimbursement programs for LLT (10).

Another important factor affecting LDL-C goal attainment is the type and competence of healthcare professionals taking care of dyslipidemia (21, 22). There are numerous reasons for the underuse (which is still unfortunately very high in Europe) of lipid-lowering drugs including clinical inertia and overuse of the diagnosis of statin intolerance (23). On the other hand, proper drug and dose adjustment is crucial to be effective in LDL-C goal achievement, and for the high to extremely high CVD risk patients, application of the upfront combination therapy, preferably with the fixed dose combination was suggested (15, 24, 25). In this analysis, despite the higher CVD baseline risk of patients from CEE countries, due to therapeutic decisions, the treatment result was as effective as (or even numerically better) in lower risk population from the remaining countries. Moreover, patients from the CEE group achieved better numerical results of LDL-C at the end of the study. This finding is probably caused by channeling bias; patients with worse baseline cardiovascular risk (and higher baseline level of LDL-C) are more likely to be given stronger and higher doses of drugs. Our results support this thesis showing that the decision of physician regarding drug and dose adjustment was based on the LDL-C level, which was true and significant for the whole cohort and Western Countries plus Canada. It seems that the decision to start a more intensive dose of alirocumab (150 mg Q2W) was not entirely based on high LDL-C levels, which may be a good sign considering that far fewer patients being on the LDL-C goal in comparison to the patients from the Western Europe (10, 26). Based on these results, it is difficult to univocally explain why LDL-C was not a significant factor for the dose adjustment in CEE countries. There might be at least few explanations, first associated with the fact that only 13% of very high-risk patients are on the LDL-C target (10)and such knowledge might have enhanced the physician's attitude to be more effective in intensive lipid-lowering therapy with innovative treatment; another one might have been a fact of very limited accessibility of PCSK9 inhibitors in the region (mainly within the reimbursement/drug program for highly selected group of patients) (6, 27). However, the real reasons of this difference should be still a matter of future investigations.

On the other hand, this study, as well as available RWE studies, also showed that even with the less experience and worse accessibility to the innovative therapies in comparison to our colleagues from Western Europe, in CEE countries, the physicians are prone to use high doses of PCSK9 inhibitors to achieve the LDL-C goal (28).

This analysis has some limitations including the possible introduction of bias as a result of the open-label design of the study. Another limitation is not the equal sample size of studied groups, which may limit the interpretation of the results. It is also worth mentioning that for the subjects with triglycerides >4.5 mmol/l (400 mg/dl), the LDL-C value was not calculated, and therefore, they were not included in the final analysis, what might have reduced the number of study participants. However, the study started in June 2015, and currently well-recognized Martin–Hopkins or Samson equations (6, 13) were not validated enough then to be applied in this study.

In conclusion, this subanalysis of the ODYSSEY APPRISE study revealed that despite previously described regional differences in the lipid-lowering efficacy, and significant differences in the use of non-statin therapy, especially with ezetimibe, and the accessibility to the PCSK9 inhibitor therapy, resulted in no significant differences in the percentage of patients being on the goal of <55 mg/dl and the combined goal of <55 mg/dl and 50% LDL-C reduction between CEE and other countries, with numerically better results for CEE patients at the end of the study. This might be the effect of more physicians who are prone to use higher doses of alirocumab, and significantly many decide to increase the dose, which ultimately is associated with a higher ratio of patients achieving the LDL-C target. The only factor that significantly influenced the decision on alirocumab dose increase/decrease was LDL cholesterol, which is however less important for physicians in CEE countries.

## Data Availability

The raw data supporting the conclusions of this article will be made available by the authors, without undue reservation.

## Data sharing

Access to patient-level data and associated study documentation, such as the clinical study report, study protocol with any revisions, blank case report form, statistical analysis plan, and data set specifications, is available upon request from qualified researchers. To preserve the privacy of trial participants, patient-level data will be anonymized, and study papers redacted. More information can be found on Sanofi's data sharing requirements, eligible studies, and the access request procedure at https://www.clinicalstudydatarequest.com.
